# Polydatin Glycosides Improve Monocrotaline-Induced Pulmonary Hypertension Injury by Inhibiting Endothelial-To-Mesenchymal Transition

**DOI:** 10.3389/fphar.2022.862017

**Published:** 2022-03-18

**Authors:** Xing Chen, Yao He, Zhijie Yu, Jianli Zuo, Yan Huang, Yi Ruan, Xiaoyuan Zheng, Yu Ma

**Affiliations:** ^1^ Pharmacy Department, Chongqing Emergency Medical Center, Chongqing, China; ^2^ Pharmacy Department, Chongqing University Central Hospital, Chongqing, China; ^3^ Chongqing Emergency Medical Center, Chongqing, China

**Keywords:** polydatin, pulmonary arterial hypertension, EndMT, HIF-2α, Arg1

## Abstract

**Objective:** To study the effect of polydatin on the injury of pulmonary arterial hypertension (PAH) induced by monocrotaline (MCT).

**Methods:** SD rats were induced to develop PAH injury by a single subcutaneous injection of MCT (60 mg/kg). From the second day, rats in the administration group were orally given sildenafil (20 mg/kg) and polydatin (30 or 60 mg/kg) for 3 weeks. At the end of the experiment, right ventricular hypertrophy (RVH) index of SD rats was calculated, pathological damage was assessed by HE staining, transcription levels of target genes were detected by RT-PCR and Elisa, and expression levels of Endothelial-to-mesenchymal transition (EndMT) related proteins were detected by immunohistochemistry (IHC) and immunofluorescence (IF). Finally, molecular docking analysis was used to verify the interaction of polydatin on the main targets.

**Results:** Polydatin could significantly restore the body function, reduce MCT-induced PAH injury, reduce serum biochemical indices; polydatin could effectively inhibit EndMT process by decreasing the expression of N-cadherin, β-catenin and vimentin; polydatin could down-regulate TAGLN expression and increase PECAM1 expression to reduce pulmonary vascular remodeling. The interaction between polydatin and EndMT target was confirmed by molecular docking operation.

**Conclusion:** Pharmacological experiments combined with Combining molecular docking was first used to clarify that polydatin can reduce the pulmonary endothelial dysfunction and pulmonary vascular remodeling induced by MCT by inhibiting EndMT. The results of the study provide new ideas for the further treatment of PAH injury.

## Introduction

Pulmonary hypertension (PAH) is still a progressive disease that seriously threatens the lives of patients. The initial symptoms of PAH are shortness of breath, fatigue and angina. With the increase of pulmonary vascular resistance, the load of right ventricle (RV) increases, which is pathologically manifested as right ventricular hypertrophy, pulmonary endothelial dysfunction and pulmonary vascular remodeling, and ultimately leads to right ventricular failure and death (del Valle and DuBrock, 2021). According to the latest global epidemiological research, the incidence of PAH are crudely estimated at 5 cases per million adults (Hoeper et al., 2016). Current therapeutic drugs, such as prostacyclin, endothelin, and nitric oxide pathways, mainly focus on improving vasodilator properties and improving cardiopulmonary function, but cannot prevent or reverse the development of PAH. The 1-year, 3-years, and 5-years survival rates of PAH patients receiving drug therapy are 85, 68, and 57%, respectively (Benza et al., 2012; Zheng et al., 2020). Therefore, exploring and elucidating the pharmacotherapy of PAH injury has profound significance for greatly reducing the incidence of PAH.

Within the settings of cardiovascular biology, Endothelial-mesenchymal transition (EndMT) plays a role in various diseases, including valvular heart disease, myocardial fibrosis, myocardial infarction and atherosclerosis. EndMT is also gradually implicated in the development and progression of PAH. Long-term chronic pressure and the internal environment of inflammatory mediators can trigger EndMT of endothelial cells. Specifically, it obtains mesenchymal cell markers, loses endothelial marker proteins (VE-cadherin, PECAM1, TIE1, and TIE2), and gains migration and invasion capabilities, ultimately contributing to the formation of obstructive intimal lesions (Good et al., 2015; Ranchoux et al., 2015). Vascular remodeling caused by endothelial cell proliferation disorder, endothelial barrier destruction and enhanced inflammatory cell infiltration are common features of PAH (Xue et al., 2020). A large number of studies have emphasized the important role of EndMT in the pathology of PAH, including human PAH and PAH experimental models (Van et al., 2014; Xue et al., 2020). The presence of transitional EndMT cells in the pulmonary vasculature of PAH patients, underscoring their important contribution to vascular remodeling and fibrosis (Thuan et al., 2018). Therefore, improving endothelial dysfunction and inhibiting EndoMT may become a new direction for research and treatment of PAH.

**FIGURE 1:**
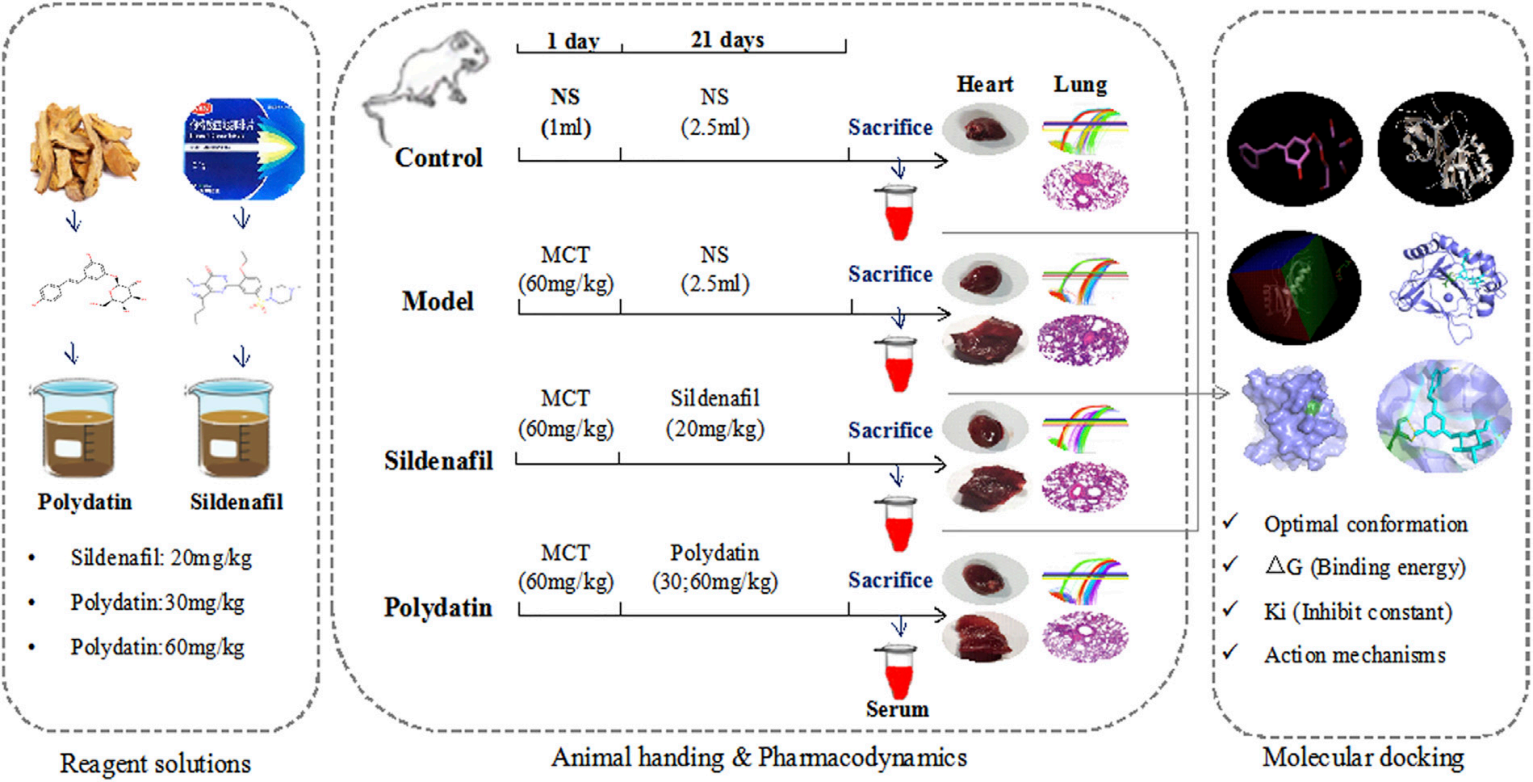
Scheme of the study.

Polydatin, a natural stilbene compound extracted from the root of polygonum cuspidatum, possesses many pharmacological activities, such as antioxidant, anti-inflammatory and improvement of microcirculation, and it has significant protective effects on lung, liver, nervous system and cardiovascular system (Zou et al., 2018; Fakhri et al., 2021; Gu et al., 2021). A large number of studies have confirmed that polydatin can play a direct vascular therapeutic role by reducing atherosclerotic vascular damage and inflammation (Peng et al., 2019; Wu et al., 2020), and it also reduce PAH in rats and improve pulmonary vascular hemodynamics against hypobaric and hypoxic PAH (Qing et al., 2009; Meng et al., 2009). Noteworthy, the latest experiments confirmed that polydatin can reduce the expression of c-Myc in human cervical cancer, inhibit cell migration and invasion, and partially reverse the EndMT of cervical cancer cells (Bai et al., 2021), and polydatin can reduce ROS and EndMT in cells exposed to high glucose, which play a therapeutic role in diabetic retinopathy (Giordo et al., 2021). Based on these findings, there is a hypothesis and tested whether polydatin can play a beneficial role in MCT-induced PAH in rats by inhibiting EndoMT and improving endothelial function (Figure 1).

## Methods

### Animals

Specified pathogen-free (SPF) male Sprague-Dawley rats (180 ± 20 g) were purchased from Weitong Lihua Laboratory Animal Technology Co., Ltd. (Beijing, China). Rats were adaptively reared in groups of 8 per cage for 1 week under certain conditions (temperature: 25 ± 0.5°C, humidity: 55 ± 5%, 12 h: 12 h light-dark cycle), and freely available food and water were provided. The animal experiment procedures in this study were carried out in strict accordance with the guidelines of the “Guidelines for the Care and Use of Laboratory Animals” of the Ministry of Science and Technology of China.

### Experimental Reagents

**FIGURE 2:**
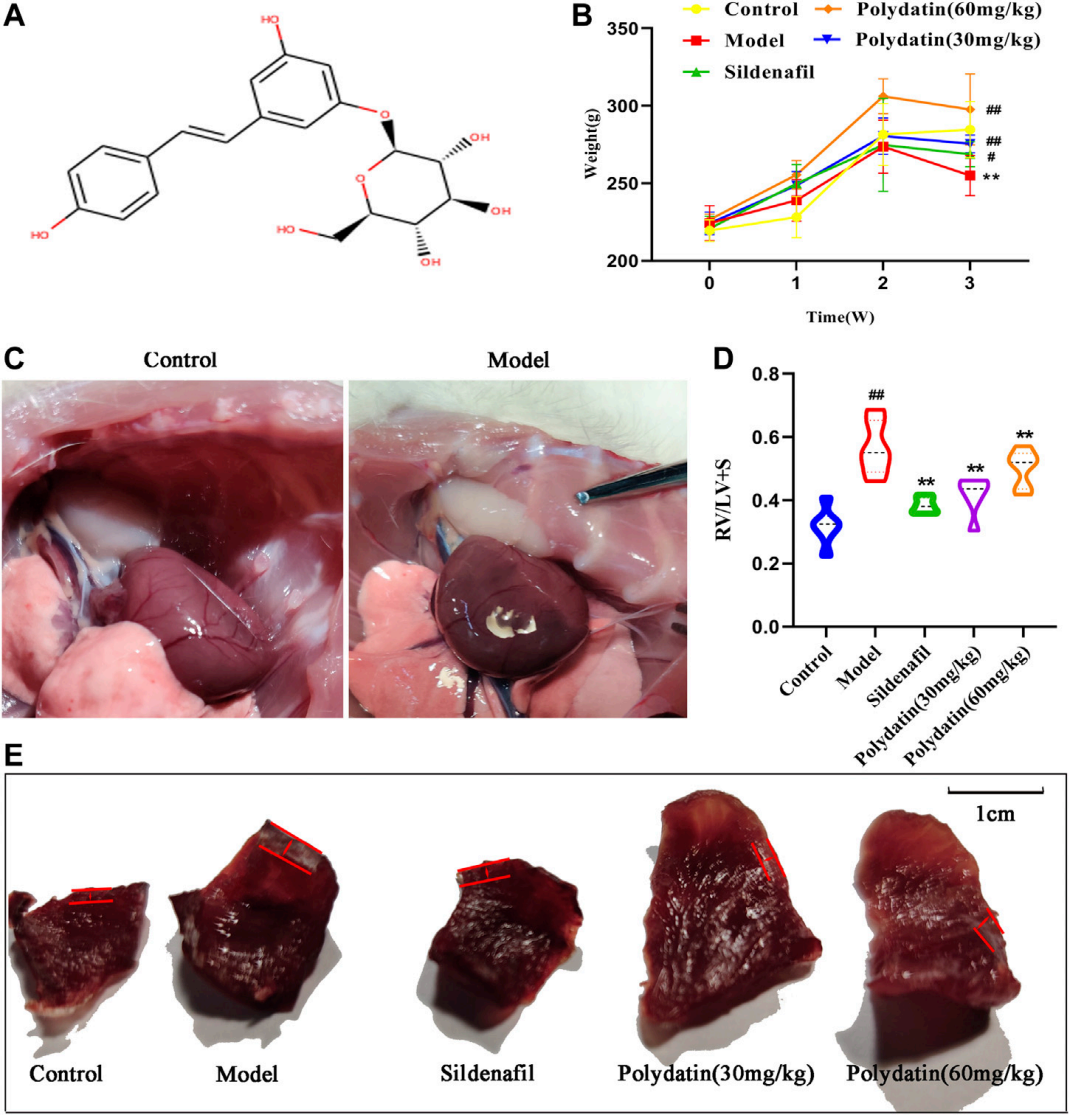
Polydatin alleviates right ventricular hypertrophy induced by MCT in SD rats. **(A)** Chemical structure of Polydatin. **(B)** Weight of SD rats (n = 6). **(C)** Morphology of rat heart. **(D)** the ratio of RV/(LV + S) (n = 6). **(E)** Morphology of right ventricular hypertrophy. Data correspond to mean values ±standard error. Groups were compared using One-way ANOVA adjusted with Dunnett’s test. *p < 0.05 and **p < 0.01 versus control group. ^#^ *p <* 0.05 and ^##^ *p <* 0.01 versus model group.

Endotoxin-free polydatin (Figure 2A, Purity ≧ 95%, CAS wkq21052105) purchased from Vikki Biotechnology Co., Ltd. (Sichuan, China). Monocrotaline (MCT) was purchased from Vikki Biotechnology Co., Ltd. (Sichuan, China). Sildenafil was purchased from Jinheng Pharmaceutical Co., Ltd. (Jilin, China). All reagents was dissolved in sodium chloride injection (NS 0.9%) and diluted to the corresponding concentration for subsequent treatment of SD rats.

### Animal Model and Experimental Protocol

The rats were randomly divided into a control group, a model group, and an intervention group. Except the control group, the other rats were injected with MCT (60 mg/kg, ig) dissolved in normal saline to induce PAH injury (Jundong et al., 2011). From the second day, rats in each group were given corresponding drugs once a day. Specifically, the control group and model group were given normal saline 2 ml/kg, the positive drug group was given sildenafil 20 mg/kg, and the paeoniflorin low-dose group Paeoniflorin was given 30 mg/kg, and the paeoniflorin high-dose group was given paeoniflorin 60 mg/kg for 3 weeks. The rats eat and drink freely throughout the experiment, and record the weight change and growth status of the rats once a week, and comprehensively evaluate the degree of the model and the therapeutic effect after the last intragastric administration. After confirming the success of the experiment, the rats were injected with 10% chloral hydrate for euthanasia, and blood samples, lungs and heart tissues were collected.

### Histopathological Examination

The isolated lungs were fixed and stored in 10% neutral formalin buffer, then embedded in paraffin to prepared into 5 μm thick sections. Hematoxylin-eosin (H&E) staining was performed according to standard procedures to assess the degree of pathological damage, then calculated the Ratio of Pulmonary Vascular Remodeling (PVR) = Vascular Wall area/total vascular area.

### Right Ventricular Hypertrophy Detection

The isolated rat heart retains the ventricular tissue after removing the atrium. Separate the right ventricle and the left ventricle + septum along the interventricular groove, and after the filter paper absorbs the water, weigh the right ventricle (RV), left ventricle (LV), and diaphragm (S) respectively, and calculate the right ventricle hypertrophy index (RVHI) = RV/(LV + S).

### Real-Time Quantitative PCR for mRNA Expressions

RNA extracts from frozen rat stomach tissues were used for microarray analysis. According to the manufacturer’s protocol, total RNA was isolated using TRIzol reagent (Nordic Bioscience, Beijing,China) and converted into cDNA using a reverse transcription kit (Promega, Madison,United States). The primer sequences of BMPR2, PHD2, HIF-2α, Arginine1 (Arg1) are shown in Table 1. Add SYBR Green PCR Master Mix (Nordic Bioscience, Beijing,China) to the sample and perform RT-PCR analysis on the 7,500 fast real-time PCR system (Applied Biosystems, Foster City, CA, United States). Using GAPDH as the endogenous reference, calculate the relative amount of mRNA based on 2-^ΔΔ^CT.

**TABLE 1:** Primers sequences.

Gene	Forward primer (5-3′)	Reverse primer (5-3′)
Rat BMPR2	CAA​AGC​CCA​GAA​GAG​CAC​AGA​GG	TTG​CCA​TCC​TGC​GTT​GAC​TCA​C
Rat PHD2	TCC​GTC​ACG​TCG​ATA​ACC​CAA​ATG	CGA​AGA​ATA​CCT​CCG​CTC​ACC​TTG
Rat HIF-2α	ACT​GAG​ACA​CCT​GCC​ACC​TTC​C	CTT​GCC​ACT​CCT​GAC​CCC​TTT​TG
Rat Arg1	AGA​CCA​CAG​TAT​GGC​AAT​TGG​AAG​C	TTG​TCA​GCG​GAG​TGT​TGA​TGT​CAG
Rat CXCL12	CGC​TCT​GCA​TCA​GTG​ACG​GTA​AG	AAG​GGC​ACA​GTT​TGG​AGT​GTT​GAG
Rat CXCR4	CAG​CCT​GTG​GAT​GGT​GGT​GTT​C	CTT​GCC​ACT​CCT​GAC​CCC​TTT​TG

### Immunohistochemistry and Immunofluorescence

Paraffin sections of lung tissue were stained with polyclonal anti-N-cadherin, anti-β-catenin, anti-vimentin, anti-PECAM1 and anti-TAGLN (Table 2). Use NIS Elements imaging software version 4.0 (Olympus, Japan) to shoot and collect images at 100×, 200× magnification.

**TABLE 2:** Antibodies and other reagents.

Antibodies and reagents	Dilution	Manufacturers
For immunohistochemical staining		
Rabbit anti-human/Rat N-cadherin	1/5,000–1/20,000	Abcam
For Immunofluorescence		
Rabbit anti-Rat β-catenin	1/500	Abcam
Rabbit anti-Rat vimentin	1/250–1/1,000	Abcam
Rabbit anti-human/Rat PECAM1	1/2000	Abcam
Rabbit anti-Rat TAGLN	1/100–1/1,000	Abcam
Mouse anti-human/Rat GAPDH	1:50,000	Proteintech
Goat anti-rabbit IgG (H + L)	1:20,000	ZSGB-BIO
Goat anti-mouse IgG (H + L)	1:20,000	ZSGB-BIO

### Enzyme-Linked Immunosorbent Assay

According to the ELISA kit (MLBIO biotechnology Co., Ltd., Shanghai, China) manufacturer’s instructions, Synergy H1 Hybrid Reader (Biotech, United States) was used to determine the concentration of HIF-2α, Arg1 in rat serum. The indicated ligand concentration in the serum is calculated as pg of the ligand or ng/mL serum.

### Molecular Docking Analysis

The 3D structures of the target protein and ligand are downloaded from the PDB database (https://www.rcsb.org/) and the ZINC database (https://zinc.docking.org/).Import AutoDockTools-1.5.6 to carry out the hydrogenation of the target protein and the removal of the water base of the ligand, and convert it into the PDBQT format. Use AutoDock vina software for molecular docking. The smaller the △G (Gibbs free binding energy) and Ki (Inhibit constant) values, the stronger the binding ability to the receptor. △G < -1.2 kcal/mol or △G < -5kj/mol indicates good binding affinity. Choose the model with the lowest free energy and use PyMOL for visual analysis.

### Statistical Analysis

All measurement data were expressed as mean ± standard deviation (S.D.). One-way analysis of variance (ANOVA) was used for analysis in the SPSS software program (version 17.0; SPSS Inc., Chicago, IL, United States). The significance of the results was evaluated by the Bonferroni method. Specifically, *p <* 0.05 was considered statistically significant, and *p < 0*.01 was considered very significant. All results were visualized in GraphPad Prism software (version 6.02; Inc., San Diego, United States).

## Results

### Polydatin Alleviates Right Ventricular Hypertrophy Induced by MCT in SD Rats

Right ventricular hypertrophy is a typical early injury of PAH. Previous studies have shown that one-time injection of MCT is an effective means to induce PAH injure in SD rats, and the disease will progress to severe stage by the third week. Therefore, the drug intervention was initiated in the administration group on the second day after MCT injection, and tested the disease model in the third week. During the whole experiment, MCT injection resulted in decreased activity and slow weight gain in SD rats (Figure 2B); the apex of the rat heart tissue was significantly rounded, and the right ventricular funnel was obviously bulged and congested (Figure 2C). Then, right ventricle and left ventricle + diaphragm were separated, and calculated the right ventricular hypertrophy index. The results showed that MCT injection caused right ventricular remodeling in rats; sildenafil and polydatin could effectively reduce right heart hypertrophy index (Figures 2D,E), suggesting that polydatin can significantly reduce the damage of PAH to heart, and enhance the self-regulation and protection of heart under pathological conditions, which is of great significance for maintaining circulatory function of the body.

### Polydatin Ameliorates the Pathological Damage of PAH Induced by MCT in Rats

**FIGURE 3:**
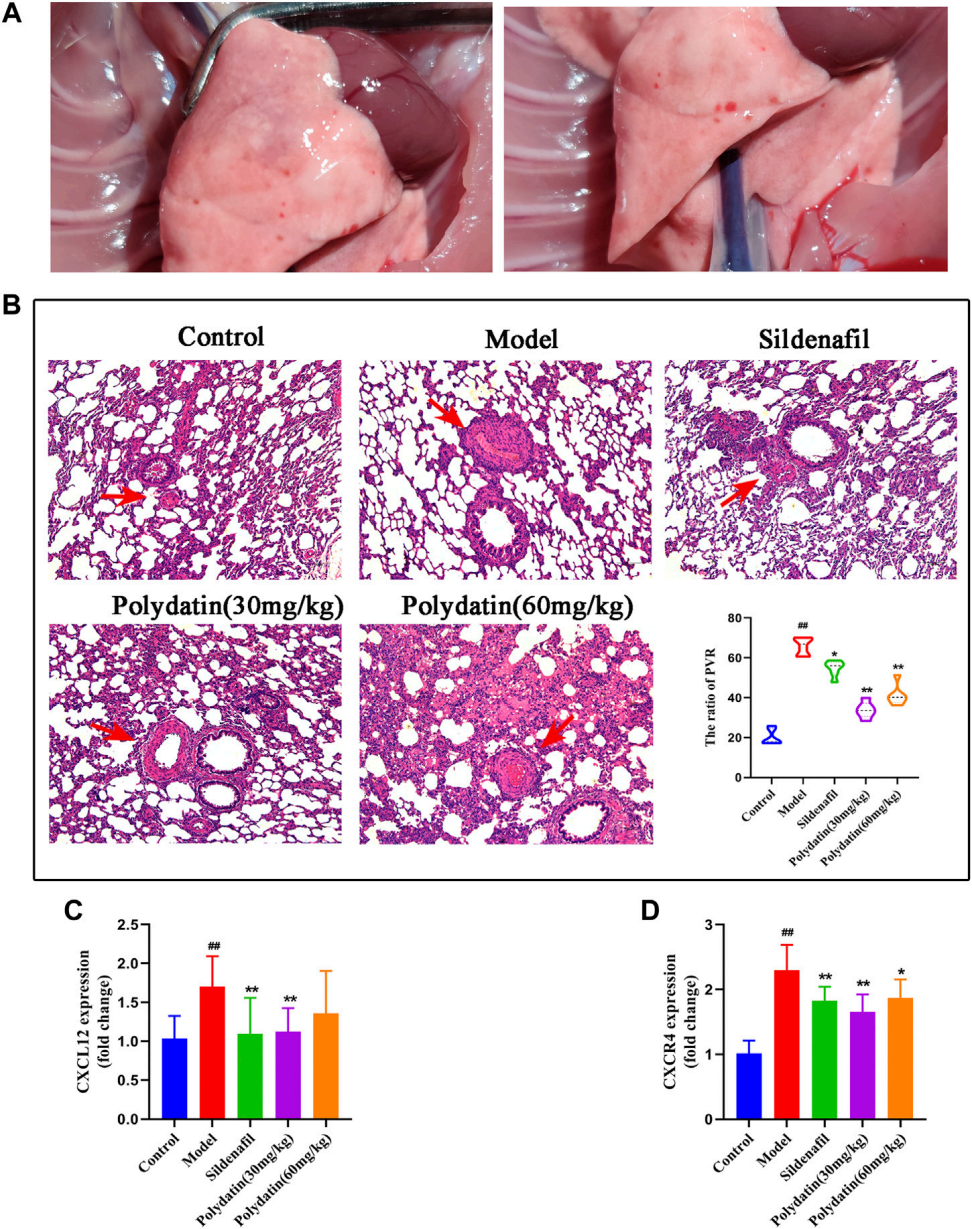
Polydatin ameliorates the pathological damage of PAH induced by MCT in SD rats. **(A)** Morphology of rat lung. **(B)** HE staining of rat lung tissue, Scale bar = 20 μm. **(C–D)** The mRNA expression levels of CXCL12 and CXCR4 (n = 6). Data correspond to mean values ±standard error. Groups were compared using One-way ANOVA adjusted with Dunnett’s test. *p < 0.05 and **p < 0.01 versus control group. ^#^ *p <* 0.05 and ^##^ *p <* 0.01 versus model group.

Consistent with clinical patients, progressive damage from PAH eventually accumulates in the lungs. In this study, injection of mct resulted in the formation of pleural and ascites in some rats, and the lungs were accompanied by congestive star spots (Figure 3A), indicating that MCT injection caused significant lung injury in SD rats. HE staining showed that the inner elastic membrane of the small pulmonary arteries of the model group was wavy, the distance between the inner and outer elastic fiber membranes was obviously widened, the thickness of the tube wall increased, the diameter of the blood vessel became smaller, and the inflammatory cells around the blood vessel showed significant Infiltration, indicating that MCT causes pulmonary vascular endothelial damage in rats, and the ratio of PVR in the model group was higher; after the intervention of sildenafil and polydatin, vascular remodeling and inflammatory cell infiltration were significantly relieved compared with the model group, and pulmonary vascular remodeling was effectively improved (Figure 3B), suggesting that polydatin can reduce the lung tissue damage induced by MCT in rats with PAH.

Inflammation also plays an important role in the process of MCT induced PAH. MCT induced pathological observation of the model showed that there were a large number of inflammatory cells infiltrated in the lung tissue after modeling, mainly distributed around the blood vessels. The pro-inflammatory factor CXCL12 and its receptor CXCR4 were significantly increased, similar to the performance of vascular inflammation. After treatment with positive drugs and polydatin, inflammatory cell infiltration and inflammatory factors were significantly improved (Figures 3C,D), indicating that polydatin can effectively alleviate the vascular inflammation induced by MCT.

### Polydatatin Inhibits MCT-Induced Activation of HIF-2ɑ/Arg1 Signaling Pathway

**FIGURE 4:**
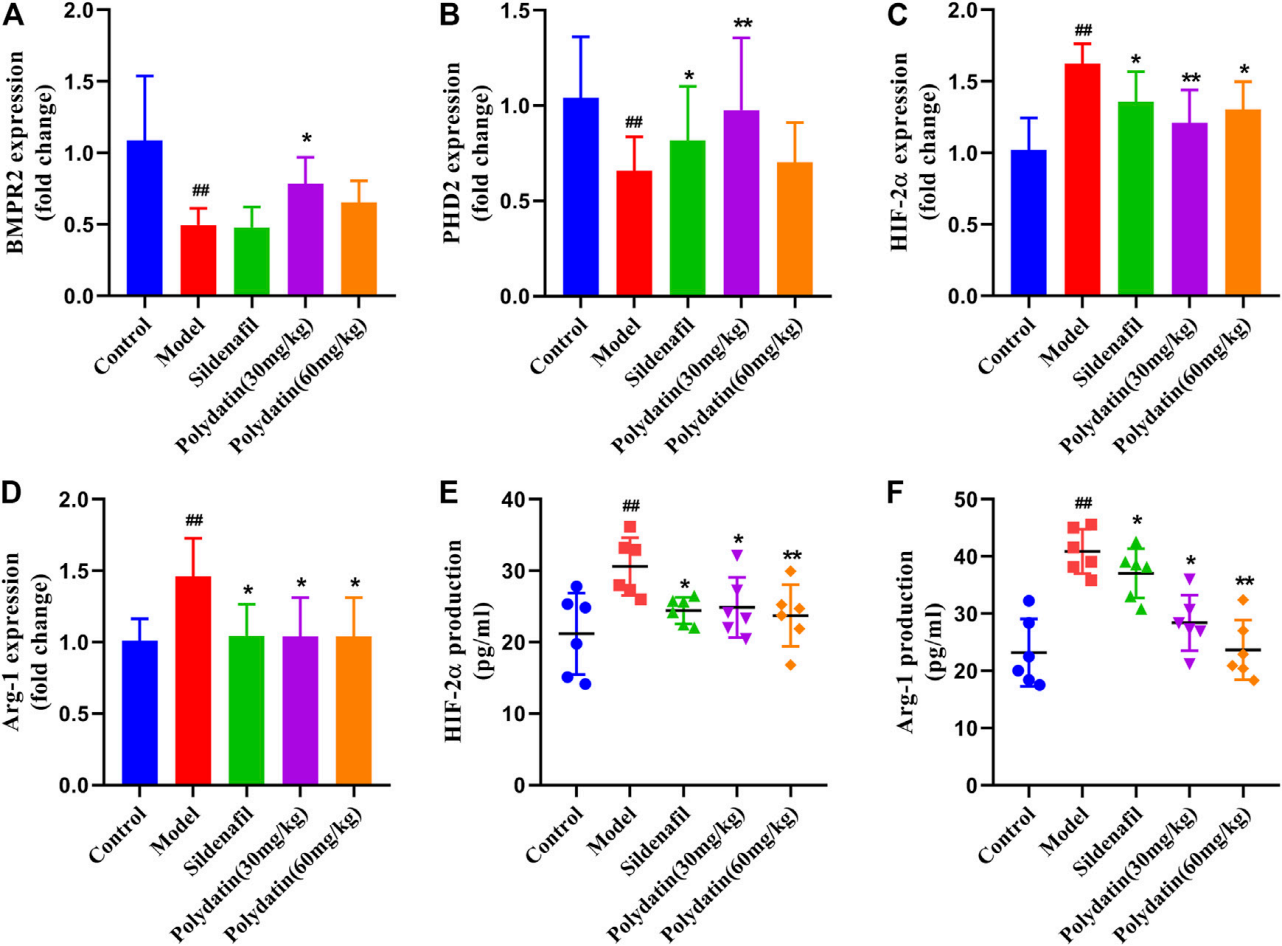
Polydatatin inhibits MCT-induced activation of HIF-2α/Arg1 signaling pathway. **(A–D)** The mRNA expression levels of BMPR2, PHD2, HIF-2ɑ and Arg1 (n = 6). **(E–F)** HIF-2ɑ and Arg1 expression and production was measured by ELISA (n = 6). Data correspond to mean values ±standard error. Groups were compared using One-way ANOVA adjusted with Dunnett’s test. *p < 0.05 and **p < 0.01 versus control group. ^#^ *p <* 0.05 and ^##^ *p <* 0.01 versus model group.

BMPR2 is the one that has been studied and found to be most related to PAH among all genes belonging to the TGF-β family, including familial primary PAH (Rol et al., 2018). BMPR2 counteracts the abnormal activation of HIF-2α, and the activity of HIF-2α is regulated by the degradation of proline-4-hydroxylase domain (PHD) protein (Kapitsinou et al., 2016; Morikawa et al., 2019). The active HIF-2α/Arg1 axis means the development of pulmonary vascular resistance and PAH (Cowburn et al., 2016). The results showed that MCT reduced the expression of BMPR2 and PHD2 (Figures 4A,B), while the mRNA expressions of HIF-2α and Arg1 were significantly increased (Figures 4C,D). In order to verify the experimental hypothesis, the expression and release detection of HIF-2α and Arg1 in SD rat serum were further tested. The results showed that compared with the control group, MCT caused up-regulation of HIF-2α levels (Figures 4A,E similar increase in Arg1protein release (Figure 4F), suggesting a significant enhancement of HIF-2α/Arg1 signaling. The intervention of sildenafil and polydatin can restore the normal expression of BMPR2 and PHD2, and block the HIF-2α/Arg1 dependent progress of PAH.

### Polydatin Improves Lung Endothelial Cell Dysfunction Induced by MCT

**FIGURE 5:**
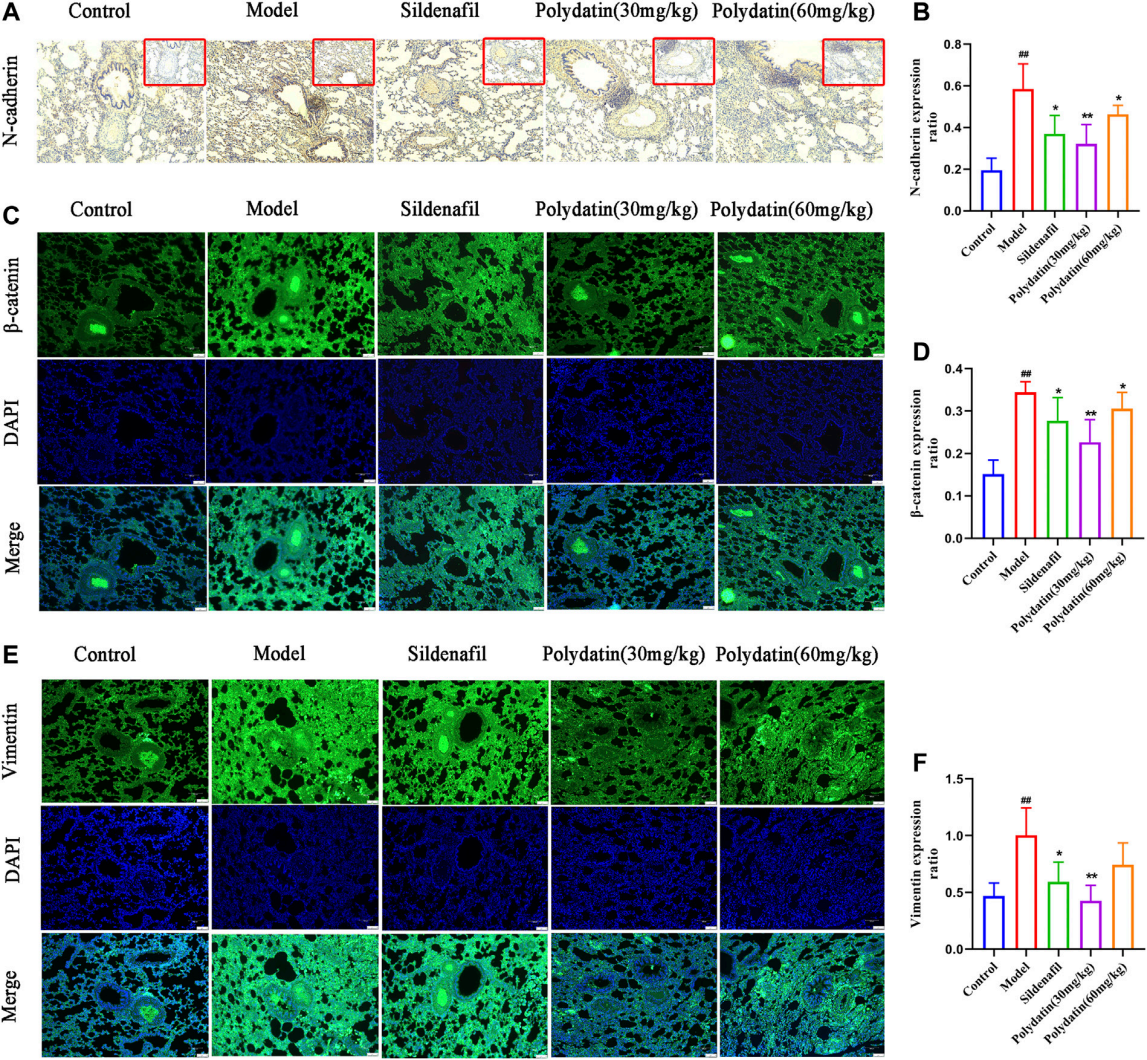
Polydatin improves lung endothelial cell dysfunction induced by MCT. **(A–B)** Representative immunohistochemical staining (100 × magnification) and gray mean values of N-cadherin expression in lung tissues. **(C–D)** Representative immunofluorescence staining (100 × magnification) and gray mean values of β-catenin expression in lung tissues. **(E–F)** Representative immunofluorescence staining (100 × magnification) and gray mean values of vimentin expression in lung tissues. Data correspond to mean values ±standard error. Groups were compared using One-way ANOVA adjusted with Dunnett’s test. *p < 0.05 and **p < 0.01 versus control group. ^#^ *p <* 0.05 and ^##^ *p <* 0.01 versus model group.

Endothelial dysfunction induced by EndMT is a contributing factor to the progression of PAH. The occurrence of EndMT breaks the tight connections between cells, causing them to lose their original stability and polarity, presenting the characteristics of loosely arranged mesenchymal cells. The results of this study showed that MCT enhanced the expression of mesenchymal cell markers in the lung tissue of the model group. For example, the expression of N-cadherin was up-regulated (Figures 5A,B), β-catenin (Figures 5C,D) and vimentin (Figures 5E,F) also showed similar strong fluorescence, suggesting endothelium the expression of intercellular connexin was inhibited. Attenuated endothelial barrier function supports higher cell migration. (Good et al., 2015). However sildenafil group and polydatin group could inhibit the expression of these mentioned mesenchymal cell markers to regain epithelial connexin, thereby preventing the migration and invasion of endothelial cells.

### Polydatin Improves Pulmonary Vascular Remodeling Induced by MCT

**FIGURE 6:**
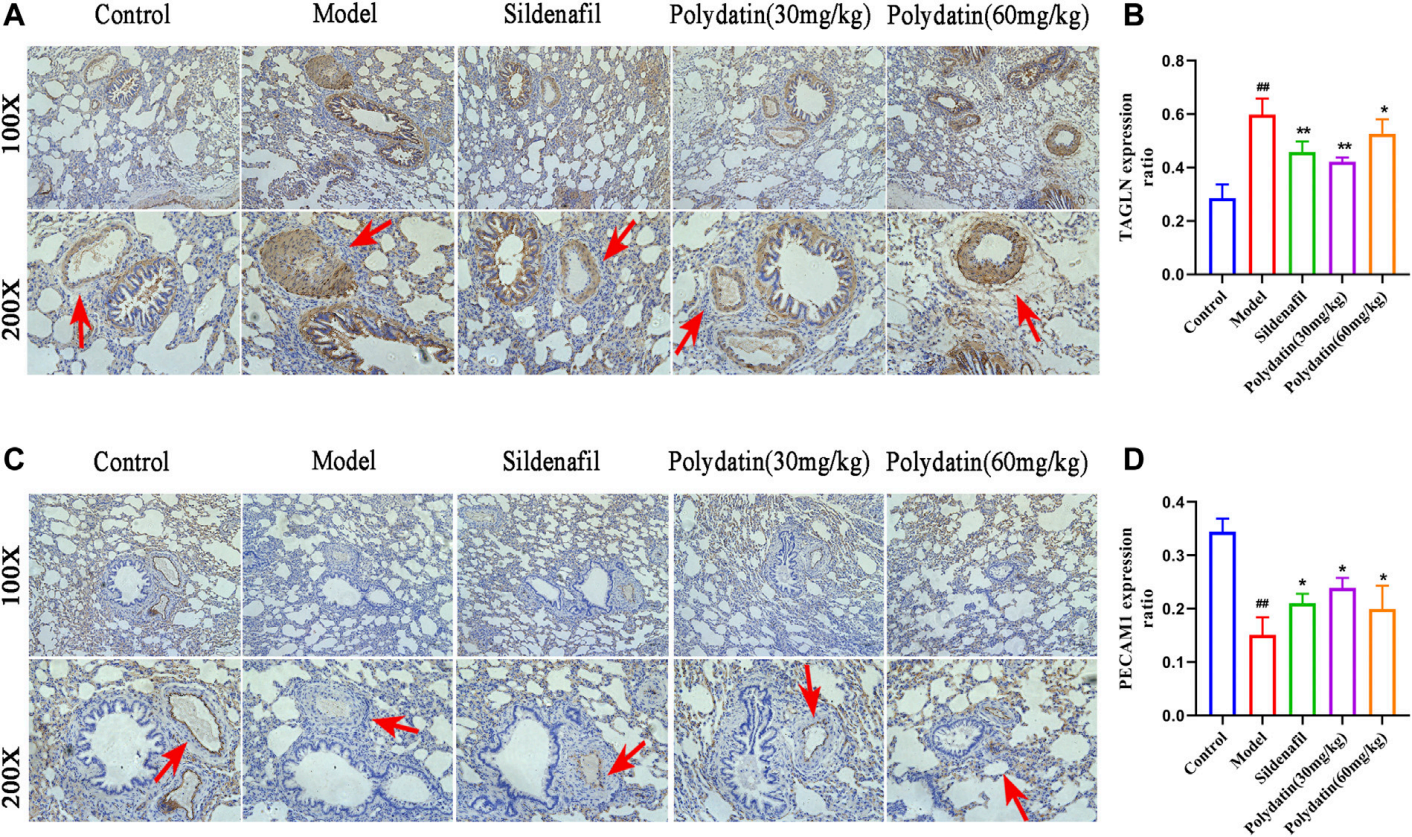
Polydatin improves pulmonary vascular remodeling induced by MCT. **(A–B)** Representative immunohistochemical staining (100 × and 200 × magnification) and gray mean values of TAGLN expression in lung tissues. **(C–D)** Representative immunofluorescence staining (100 × and 200 × magnification) and gray mean values of PECAM1 expression in lung tissues. Data correspond to mean values ±standard error. Groups were compared using One-way ANOVA adjusted with Dunnett’s test. *p < 0.05 and **p < 0.01 versus control group. ^#^ *p <* 0.05 and ^##^ *p <* 0.01 versus model group.

EndMT is an important mechanism of pulmonary vascular remodeling in animal PAH models and human PAH patients. Injured pulmonary vessels can trigger endothelial cell muscularization, which further leads to pulmonary arterial wall thickening and even the formation of occlusive neointima, representing an irreversible stage in the pathology of pulmonary hypertension. PECAM (ECs) cell marke and TAGLN (SMC marker) have been used to assess reendothelialization rate of endothelial cells in several cardiovascular disease studies (Hutter et al., 2003; Ouyang et al., 2021). This study showed that MCT-induced EndMT further promoted the increase in the expression of TAGLN in intraluminal obstruction in model group (Figures 6A,B), while the expression of PECAM1 (CD31) decreased or even lost (Figures 6C,D), suggesting the formation of pulmonary artery muscle tissue and neointima in model group; while the sildenafil and polydatin maintained the above-mentioned protein expression at a normal level, indicating that polydatin can effectively alleviate the pulmonary vascular remodeling induced by MCT.

### Molecular Docking Analysis of Polydatin to Key Targets of PAH

Pharmacological studies have shown that polydatin can inhibit EndMT triggered pulmonary endothelial dysfunction and pulmonary vascular remodeling, which may be mediated by inhibiting upstream targets. Therefore, molecular docking of polydatin with BMPRR2, PHD2, HIF-2α and Arg1 was performed to investigate whether polydatin is an effective inhibitor of PAH disease progression.

**FIGURE 7:**
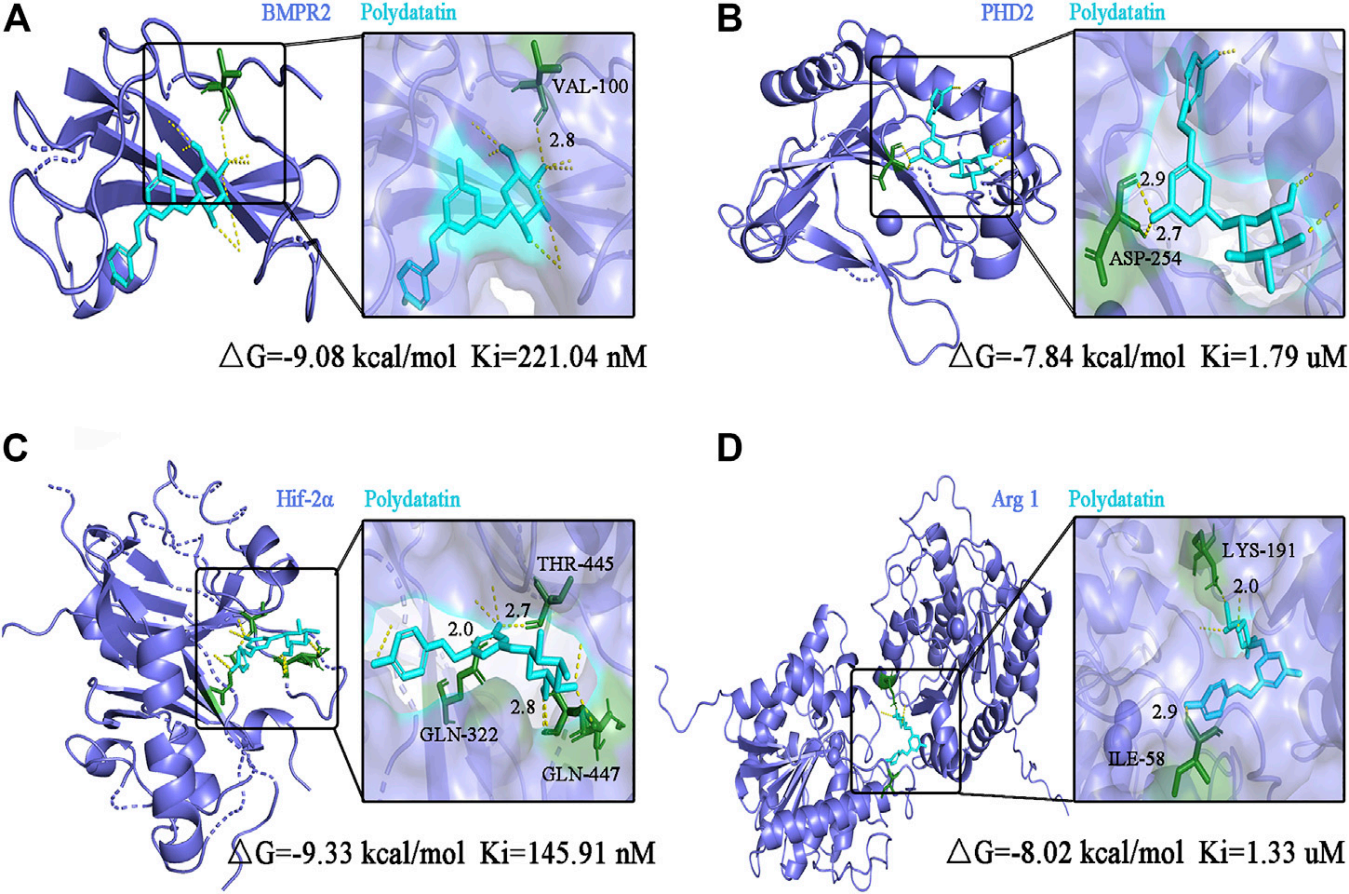
Molecular docking analysis of polydatin to key targets of PAH induced by MCT. **(A–D)** Complex model of polydatin to human BMPR2 **(A)**, PHD2 **(B)**, HIF-2α **(C)** and Arg1 **(D)** rendered in backbone cartoon (left) or in molecular surface (right).

Figure 7 shows the conformation of the molecular docking experiment. Only the optimal docking between the compound and the active center site of the protein was screened and labeled. The hydrogen bonds were highlighted by yellow dotted lines, and dark green represented the amino acid residues closely linked to the compound in the active site of the protein, and finally marked the interatomic distance. Molecular docking calculation showed that VAL-100 of BMPR2 interacted with polydatin via hydrogen bond (△G = -9.08 kcal/mol Ki = 221.04 nM) (Figure 7A). ASP-254 of PHD2 interacts with polydine through hydrogen bond (△G = -7.84 kcal/mol Ki = 1.79 uM) (Figure 7B). THR 445, GLN-322 and GLN-447 of HIF-2α interact with polydatin via hydrogen bond (△G = −9.33 kcal/mol Ki = 145.91 nM) (Figure 7C). Lys-191 and ILe-58 of Arg1 interact with polydine through hydrogen bonds (△G = −8.02 kcal/mol Ki = 1.33 uM) (Figure 7D). The four protein targets showed good docking results with polydatin (△G < −1.2 kcal/mol). The Ki value of BMPR2 and HIF-2α docking with polydatin was lower than that of PHD2 and Arg 1, and even reached nanomolar level. Therefore, BMPR2, PHD2, HIF-2α and Arg1 proteins may be the main targets of polydatin against PAH injury.

## Discussion

**FIGURE 8:**
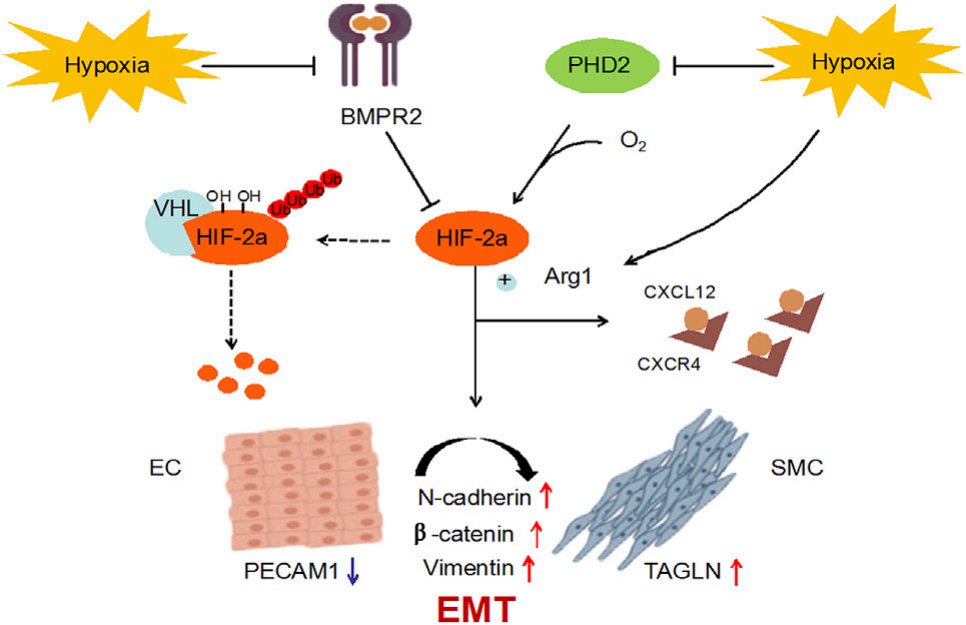
Polydatin improves MCT-induced PAH by inhibiting EndMT.

Polydatin is the product of the combination of resveratrol and glucose, also known as resveratrol glycosides. Polydatin and resveratrol have similar pharmacological effects, and they can be interconverted *in vivo* (Wang and Zhang, 2017). Polydatin tends to be more abundant than resveratrol in nature (Song et al., 2019). Therefore, many studies have been devoted to the conversion of polydatin to resveratrol, but the functional properties of polydatin with stronger antioxidant effects and metabolic stability have been ignored (Platella et al., 2019). Among the numerous pharmacological effects, antioxidant effect is undoubtedly the most important, and vascular damage caused by reactive oxygen species (ROS) also plays an important role in cardiovascular diseases (Krzemińska et al., 2022). Therefore, the biological properties and various pharmacological effects of polydatin make it have high research and application value, and it is expected to become a characteristic new drug for the prevention and treatment of cardiovascular diseases. This study confirmed for the first time that resveratrol glycosides can inhibit HIF-2α/Arg1 signaling by inhibiting the protein expression of BMPR2 and PHD2, thereby improving MCT-induced pulmonary endothelial dysfunction and pulmonary vascular remodeling (Figure 8). It is worth noting that the effect of high dose of polydatin in improving the pathological damage of PAH is inferior to that of low dose, and it has a slight pro-fibrotic effect. It is possible that polydatin at high concentration activates the pro-fibrosis factor (Liu et al., 2019), which damages its anti-fibrosis effect and aggravates lung injury induced by MCT. Therefore, the benefits of polydatin are varied and highly dose-dependent, but the mechanism by which high doses promote pulmonary fibrosis remains to be explored.

BMPR2 mutation is a key risk factor for hereditary pulmonary arterial hypertension (hPAH), and about 20% of carriers will get the disease (Thomson et al., 2000). The importance of BMPR2 dysfunction in PAH is supported by research in transgenic mice, and human patients also show more severe pulmonary vascular remodeling (Stacher et al., 2012). HIF is a key regulator of transcription factors and molecular responses to hypoxia. HIF-2α, as the direct target of BMPR2, is the mediating hub that regulates pulmonary vascular response. Transcription analysis of PAH-related gene expression suggests that HIF-2α mediates the differential expression of a large number of genes (Zhu et al., 2021). Prolyl hydroxylase domain protein (PHD) is the most important isoenzyme under normoxic conditions and is involved in a variety of hypoxic stress processes, such as angiogenesis and cardiac function. PHD2 can hydroxylate the conserved proline residues in HIF-2α and further mediate the degradation of the complex between von Hippel-Lindau protein (VHL) and HIF-2α. Therefore, BMPR2 and PHD2 together affect the protein abundance and activity of HIF-2α. Arg1, a HIF-2α dependent gene, is down-regulated in HIF-2α pulmonary endothelial mutants, leading to a reduction in NOS source and NO formation (Cowburn et al., 2016). In this study, the expression of PAH-related genes was abnormal after injection of MCT in SD rats, which indicated that MCT caused progressive damage to PAH in rats; After the intervention of polydatin, the expression levels of the above genes could be significantly recovered, suggesting that polydatin could resist MCT induced lung disease injury.

A complete pulmonary endothelial barrier is indispensable for maintaining vascular homeostasis. EndMT is an important process for cells to acquire mesenchymal properties and movement, and plays a key role in the progression of the disease. N-cadherin and vimentin are considered to be typical mesenchymal markers, which are usually used to reflect the progress of EndMT (Ranchoux et al., 2015). β-catenin binds and dissociates cytoskeleton proteins to promote cell migration by regulating cytoskeleton and intercellular co-adhesion (Zhang et al., 2017). In this study, MCT induced up-regulation of N-cadherin, vimentin and β-catenin in lung tissue of SD rats, suggesting that endothelial injury triggered endothelial cell proliferation and migration to restore endothelial barrier integrity and vascular homeostasis. Polydatin could effectively maintain the integrity of the cell-cell connection complex and reduce the expression of mesenchymal markers.

Progressive pulmonary vascular remodeling is one of the main causes of disease progression in almost all PAH patients. A series of studies relying on autopsy samples of severe PAH have emphasized pulmonary vascular alterations, showing pulmonary endothelial cell growth disorder, leading to plexiform lesions (Wagenvoort et al., 1970; Zhang et al., 2017). Muscularization after endothelial cell injury is an important factor in determining the size of neovascularization (Tuder et al., 2007). In this study, MCT induced pulmonary vascular endothelial cells (EC) to undergo EndMT, which showed that EC cells acquired the smooth muscle cell (SMC) marker (up-regulated in TAGLN expression) and lost the EC marker (down-regulated in PECAM1 expression), suggesting that PAH-related stress promoted the formation of pulmonary vascular neointima in SD rats. As expected, polydatin effectively prevented phenotypic transformation of pulmonary endothelial cells and reversed PAH-associated pulmonary vascular remodeling.

Perivascular inflammation also plays an important role in vascular remodeling and ultimately drives the progression of PAH (Rabinovitch et al., 2014). CXCL12, also known as stromal cell-derived factor 1 (SDF1), is an angiogenic chemokine that acts by binding to its homologous receptor CXCR4 or CXCR7. CXCL12 promotes the formation of new blood vessels in multiple organs, including the development of skeletal muscle and heart arteries, while promotes tumor and leukemia progression and accelerates atherosclerosis under pathological conditions (McCullagh et al., 2015; Yi et al., 2021). Earlier studies showed that CXCL12 was elevated in plasma and CXCR4 was significantly upregulated in hypoxia-induced PAH rats, and the same trend was observed in clinical samples; pharmacological inhibition of CXCR4 reversed RV hypertrophy, pulmonary artery middle layer hypertrophy and pulmonary vascular remodeling in PAH rats (Xu et al., 2021). In this study, CXCL12 was highly expressed in MCT injected SD rats, and CXCR4 was also significantly upregulated. However, CXCL12/CXCR4 was significantly inhibited after 3 weeks of treatment with polydatin, suggesting that polydatin can effectively improve perivascular inflammation induced by MCT in SD rats.

Next, molecular docking analysis confirmed the interaction of polydatin with key targets of PAH. Abnormal expression of BMPR2 and PHD2 has been included in the progression of EndMT - related diseases in early studies (Sun et al., 2020; Guan et al., 2021), which together regulated the abundance and activity of HIF-2ɑ protein. Targeting HIF2α/AGR1 is considered as a novel treatment strategy for PAH, and Arg1 overexpression has been reported to be positively correlated with the viability and invasion ability of hepatocellular carcinoma cells (You et al., 2018; Macias et al., 2021). Therefore, it is reasonable to believe that focusing on improving the EndMT process can help prevent the progression of PAH disease. The results of molecular docking operations confirmed that polydatin could deeply bound to BMPR2, PHD2, HIF-2α and Arg1 protein, and established a strong interaction network on the HIF2α/Arg1 signal axis, which effectively inhibited EndMT process and ameliorated MCT-induced PAH progressive damage.

At present, no toxicological and safety reports of polydatin have been found in literature search. More importantly, polydatin can be fully absorbed by human body through passive diffusion and active transport (Yee, 1997). Lv et al. (2006) confirmed that Wistar rats could be rapidly absorbed into the blood after orally taking 50 mg/kg of resveratrol glycoside, reaching the maximum concentration in most tissues within 10 min, and reaching the peak content in the heart 30 min later. These studies laid a foundation for the protective effect of polydatin on cardiovascular system and provided guidance for clinical practice. To investigate the ameliorative effect of polydatin on PAH injury, this study established MCT-induced PAH related injury, including right ventricular compensatory hypertrophy, CXCL12/CXCR4 dominated pro-inflammatory environment, impairment of HIF-2ɑ/Arg1 pathway, endothelial dysfunction, and vascular remodeling. Interestingly, clear associations have been reported between these disease-related states and EndMT. Next, the infection of individuals who routinely ingest polydatin from natural or supplementary sources is simulated. Data showed that polydatin significantly inhibited EndMT process, ultimately improved perivascular and interstitial inflammatory infiltration, blocked HIF-2ɑ/Arg1 signaling pathway, improved pulmonary endothelial dysfunction and alleviated pulmonary vascular remodeling.

## Conclusion

In summary, the present study supported that polydatin exerted protective effect on on MCT-induced PAH injury. Polydatin attenuates MCT-induced right ventricular compensatory hypertrophy and CXCL12/CXCR4 related inflammatory response, improves pulmonary endothelial dysfunction and inhibits pulmonary vascular remodeling. The underlying mechanism may involve inhibition of EndoMT by blocking HIF-2ɑ/Arg1 signaling. Overall, these experimental data indicate suggest that polydatin has great potential and specific therapeutic value for the development of innovative drugs to ameliorate PAH related injury.

## Data Availability

The original contributions presented in the study are included in the article/Supplementary Materials, further inquiries can be directed to the corresponding authors.
